# BTO-Coupled CIGS Solar Cells with High Performances

**DOI:** 10.3390/ma15175883

**Published:** 2022-08-25

**Authors:** Congmeng Li, Haitian Luo, Hongwei Gu, Hui Li

**Affiliations:** 1 Institute of Electrical Engineering Chinese Academy of Sciences, Beijing 100190, China; 2 University of Chinese Academy of Sciences, Beijing 100049, China

**Keywords:** thin-film solar cells, CIGS solar cells, ferroelectric materials, BaTiO_3_, SCAPS simulation

## Abstract

In order to improve the power conversion efficiency (PCE) of Cu(In,Ga)Se_2_ (CIGS) solar cells, a BaTiO_3_ (BTO) layer was inserted into the Cu(In,Ga)Se_2_. The performances of the BTO-coupled CIGS solar cells with structures of Mo/CIGS/CdS/i-ZnO/AZO, Mo/BTO/CIGS/CdS/i-ZnO/AZO, Mo/CIGS/BTO/CdS/i-ZnO/AZO, Mo/CIGS/CdS/BTO/i-ZnO/AZO, Mo/CIGS/BTO/i-ZnO/AZO, Mo/CIGS/CdS/BTO/AZO, and Mo/ CIGS/CdS(5 nm)/BTO(5 nm)/i-ZnO/AZO were systematically studied via the SCAPS-1D software. It was found that the power conversion efficiency (PCE) of a BTO-coupled CIGS solar cell with a device configuration of Mo/CIGS/CdS/BTO/AZO was 24.53%, and its open-circuit voltage was 931.70 mV. The working mechanism for the BTO-coupled CIGS solar cells with different device structures was proposed. Our results provide a novel strategy for improving the PCE of solar cells by combining a ferroelectric material into the *p*-*n* junction materials.

## 1. Introduction

Solar cells show great potential for solving the urgent energy and environmental crisis. The mainstream solar cells in the market are Si solar cells, which have more than 90% of the market share [1]. Compared with Si solar cells, thin-film solar cells, such as Cu(In,Ga)(Se)_2_ (CIGS), CdTe, and perovskite solar cells, show superior advantages in weak light effect and fabrication of flexible devices, rendering their potential applications in building integrated photovoltaic, portable application, and so on [1]. Among thin-film solar cells, CIGS solar cells have attracted intensive attention due to their high record power conversion efficiency (PCE) of 23.35% [2], easily tunable bandgap (*E*_g_) in the range of 1.04–1.68 eV, great potential applications in perovskite tandem solar cells, low cost of $0.34 W^−1^, low-temperature coefficient of −0.32%/K, and high stability [1]. However, the average PCE of a practical CIGS module is only 13–15%, which is substantially lower than the theoretical PCE of ~33.2% according to the Shockley–Queisser (SQ) limit [3,4]. The low practical PCEs are mainly ascribed to the open-circuit voltage (*V_oc_*) loss (*V_oc, loss_* = *E*_g_/*q* − *V_oc_*). The *V_oc, loss_* mainly stems from the non-radiative recombination induced by the interface and bulk defects.

Many strategies have been applied to passivate the bulk and interface recombination defects, which is beneficial to the improvement of the *V_oc_* and PCE. Nevertheless, the practical PCEs of solar cells with ideal passivation still have limitations in surpassing the theoretical PCE based on the SQ limit [5]. In contrast, the ferroelectric-coupled photovoltaic (PV) device, where a ferroelectric depolarization field, aroused by the ferroelectric materials, is coupled into the common *p*-*n* junction, shows an extremely high PCE because of the anomalously high built-in electric field of ~10^5^ V/cm [6,7,8]. The ferroelectric-coupled PV device has great potential to exceed the theoretical PCE. For this, ferroelectric materials have been widely combined into organic PV devices and perovskite solar cells. For instance, a 10 nm ferroelectric polymer film was inserted into the polymeric organic solar cell [9]. The PCE of the solar cell was increased from 1–2% to 4–5%. The improved PCE is due to the enhanced electron transport capability, which is due to the large and permanent internal depolarization field of the ferroelectric layer. Similarly, an ultrathin BaTiO_3_ (BTO) layer was inserted into the TiO_2_ and perovskite layers in the perovskite solar cell to reduce the charge recombination. The BTO layer retarded the charge recombination and improved the carrier extraction rate at the interface, thus increasing the PCE (from 16.13% to 17.87%) [10]. These results confirm that the ferroelectric-coupled solar cells show great potential for the PCE improvement by reducing the *V_oc, loss_*.

Various ferroelectric materials, such as PVDF-TrFE [8], Pb(Zr,Ti)O_3_ [11], BiFeO_3_, Bi_2_FeCrO_6_, BTO, LiNbO_3_ [12], Be*_x_*Cd_y_Zn_1-*x*-*y*_O, PbTiO_3_, and Pb_(1-*x*)_La*_x_*ZrTiO_3_ [13], have been applied to enhance the internal electric field of a solar cell by the combination of the ferroelectric depolarization field with the *p*-*n* junction field. Among these ferroelectric materials, BTO shows superior properties of a wide bandgap (*E*_g_ ~ 3.4 eV), room-temperature ferroelectricity (*T_c_* ~ 120 °C), substantial remnant polarization (*P_r_* = 0.5 C/m^2^), environmental-friendly advantage, easy fabrication, excellent stability, and so on [14,15,16,17,18,19,20]. The internal electric field of a solar cell is expected to be significantly enhanced by the insertion of a BTO ferroelectric layer into the device. The spontaneous polarization and domain structure of the ferroelectric BTO layer is used to improve the built-in electric field. The improved electric field can enhance the carrier separation and transportation and reduce the carrier recombination, which is beneficial to the improvement of the *V_oc_*, short-circuit current density (*J_sc_*), and, thus, the PCE.

Compared with other experiments, theoretical simulation provides a feasible approach to elucidate the performance and working mechanism of BTO-coupled solar cells clearly, which is helpful to the design and fabrication of practical devices. SCAPS-1D is a widely applied simulation tool to study the performance of a solar cell [21,22]. Lots of information, including the static energy band diagram, current density-voltage (*J*-*V*) curve, external quantum efficiency (EQE) curve, capacitance-voltage (*C*-*V*) curve, capacitance-frequency (*C*-*f*), carrier transportation, and recombination currents from bulk and interface defects, are easily obtained via the simulation via the SCAPS-1D software. For example, the performances of the novel Cu_2_BaSnS_4_ solar cells were well studied by the SCAPS-1D software [23,24]. The SCAPS-1D simulation indicates that an added different Back Surface Field (BSF) layer in Cu_2_BaSnSSe_3_ solar cells could increase the *V_oc_*, which is consistent with the experimental results [25]. The software is also applied to simulate and modify CIGS [21], Cu_2_ZnSnS_4_ (CZTS) [26], and perovskite solar cells [27] to study the effect of the materials and thickness of the window layer [28], buffer layer [29], and the electrode on the device performances.

SCAPS-1D is a one-dimensional solar cell simulation software. It is based on solving three non-linear differential equations: Poisson’s equation, the continuity equation for free electrons, and the continuity equation for free holes, as shown in Equations (1)–(3) [30]:

(1) $$\frac{d}{dx}\left(\varepsilon \left(x\right)\frac{d\varphi }{dx}\right)=q\left[p\left(x\right)-n\left(x\right)+N_{D}^{+}\left(x\right)-N_{A}^{-}\left(x\right)+p_{t}\left(x\right)-n_{t}\left(x\right)\right]$$

(2) $$-\frac{1}{q}\frac{dJ_{n}}{dx}+R_{n}\left(x\right)-G\left(x\right)=0$$

(3) $$\frac{1}{q}\frac{dJ_{p}}{dx}+R_{p}\left(x\right)-G\left(x\right)=0$$

where *ψ*, *n*, *p*, *n_t_*, *p_t_*, *N_D_*^+^, and *N_A_*^−^ are the electrostatic potential, free electrons density, free holes density, electrons distribution, holes distribution, ionized donors’ concentration, and ionized acceptors concentration, respectively. *R_n_*(*x*), *R_p_*(*x*), *G*(*x*), *J_n_*, *J_p_*, *ε,* and *q* are the electrons recombination rate, holes recombination rate, generation rate, electron current density, hole current density, permittivity, and charge of the electron, respectively [24].

In this study, a BTO ferroelectric layer was incorporated into the CIGS solar cell. The impact of the BTO layer on the performance of the CIGS solar cell was systematically investigated via the SCAPS-1D software. BTO-coupled CIGS solar cells with various device structures were simulated. Our results show that the optimized device architecture is: Mo/CIGS/CdS/BTO (5 nm)/AZO with a PCE of 24.53% and a *V_oc_* of 931.70 mV. The combination of a ferroelectric layer into a common *p*-*n* junction solar cell is helpful for the improvement of the device’s performance. Our work provides a novel device structure for the PCE improvement of the CIGS solar cell by the incorporation of a ferroelectric layer into the device structure. This strategy is also applicable to other solar cells.

## 2. Methodology

**Figure 1:**
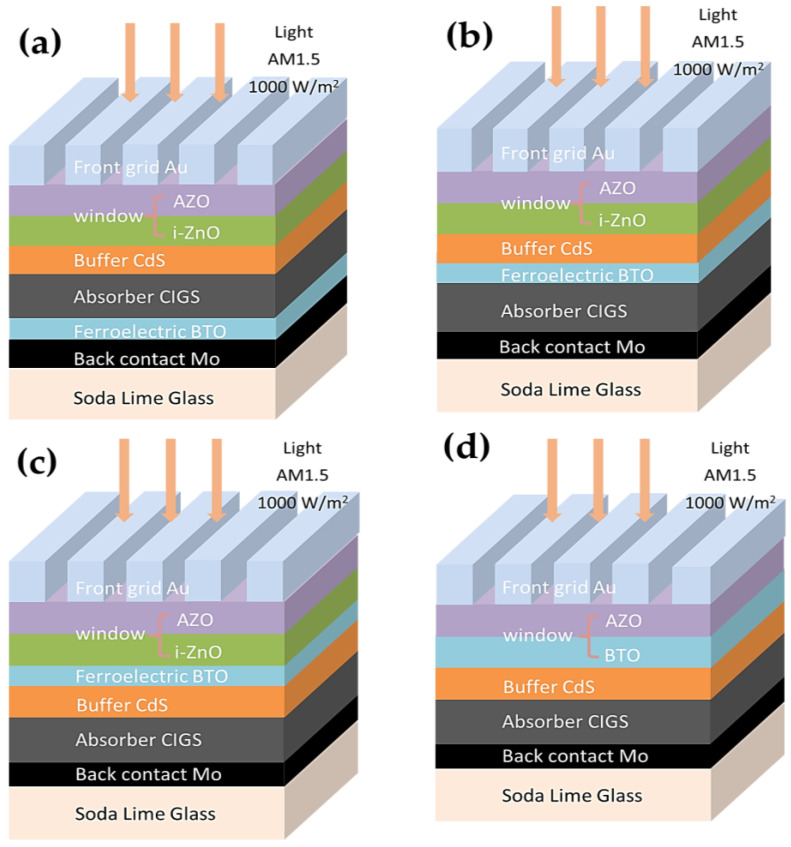
Device architectures for simulation: (**a**) SLG/Mo/BTO/CIGS/CdS/i-ZnO/AZO/Au, (**b**) SLG/Mo/CIGS/BTO/CdS/i-ZnO/AZO/Au, (**c**) SLG/Mo/CIGS/CdS/BTO/i-ZnO/AZO/Au, (**d**) SLG/Mo /CIGS/CdS/BTO/AZO/Au.

**Figure 2:**
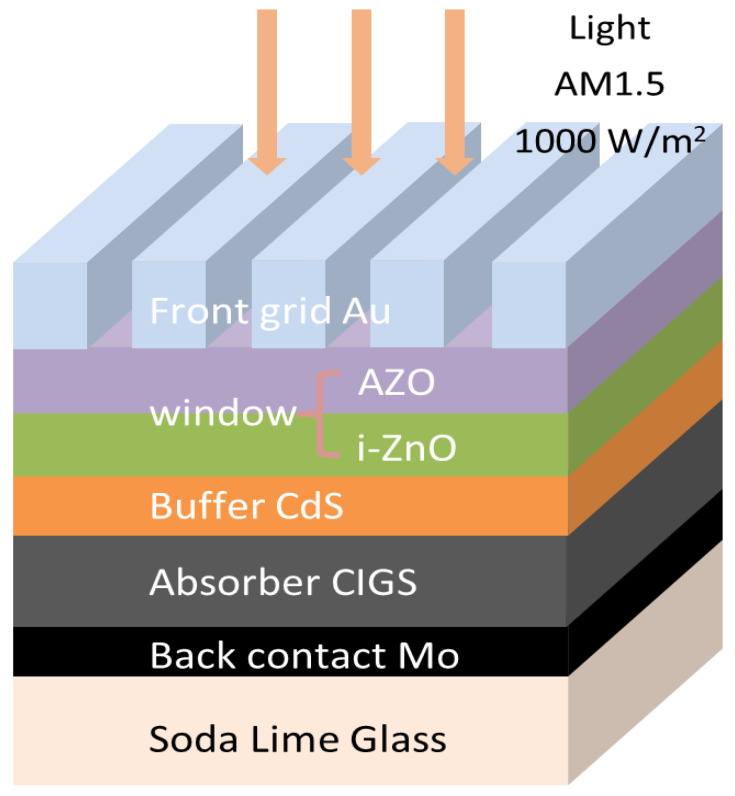
Device architecture of the simulated CIGS solar cell.

In order to elucidate the impact of a BTO layer on the performance of the CIGS solar cell, four device configurations were proposed and simulated: soda-lime glass (SLG)/Mo/BTO/CIGS/CdS/i-ZnO/Al-doped zinc oxide (AZO)/Au (Figure 1a), SLG/Mo/CIGS/BTO/CdS/i-ZnO/AZO/Au (Figure 1b), SLG/Mo/CIGS/CdS/BTO/i-ZnO/AZO/Au (Figure 1c), and SLG/Mo/CIGS/CdS/BTO/AZO/Au (Figure 1d). For comparison, the performance of the CIGS solar cell with a typical device configuration of SLG/Mo/CIGS/CdS/i-ZnO/AZO/Au (Figure 2) was also simulated. Note that the BTO was easily polarized under an external field, which shows that the depolarization field is induced by the *p*-*n* junction field during the simulation.

The parameters for different layers in the simulated devices are listed in Table 1. The *J*-*V* curves, EQE curves, *J_sc_*, *V_oc_*, fill factor (FF), and PCEs for the simulated devices were obtained under the condition of AM1.5 G, an incident solar power (*P*) of 100 mW/cm^2^, and a temperature of 300 K. In addition, the band diagram and *C*-*V* curves were also obtained.

## 3. Results and Discussion

### 3.1. Impact of CIGS Bandgap on Performances of CIGS Solar Cells

**Figure 3:**
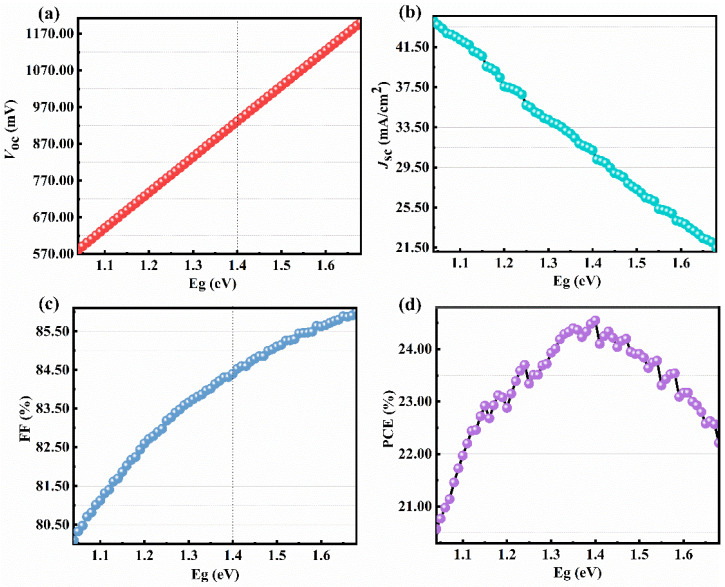
Dependence of (**a**) *V_oc_*, (**b**) *J_sc_*, (**c**) FF, and (**d**) PCE of CIGS solar cells on CIGS bandgap.

It is well known that the PCE of a solar cell is closely related to the bandgap of the absorbing layer. Only photons with an energy *hν* higher than the optical bandgap of the absorbing layer can be absorbed. The bandgap of CIGS is well modified in the region of 1.04–1.68 eV by the modification of the In and Ga atomic ratio. Therefore, it is especially important to clarify the impact of the bandgap of CIGS on the device’s performance. For this, the CIGS solar cell with a typical structure of SLG/Mo/CIGS/CdS/i-ZnO/AZO/Au (Figure 2) is firstly investigated via the SCAPD-1D software. The program is developed at the department of Electronics and Information Systems of the University of Gent, Belgium. The version number we use is SCAPS 3.3.07, and it can be freely available. As shown in Figure 3, the *V_oc_*, *J_sc_*, FF, and PCE show a close relationship with the bandgap of CIGS. The *V_oc_* linearly increases with the bandgap of CIGS (Figure 3a). It is well known that the *V_oc_* can be calculated based on the following equations [34,35]:

(4) $$V_{oc}=\frac{kT}{q}\ln\left(\frac{J_{sc}}{J_{0}}+1\right)$$

(5) $$J_{0}\propto n_{i}^{2}=N_{C}N_{V}\exp\left(-\frac{E_{g}}{kT}\right)I_{0}=1.5\times 10^{5}\exp\left(-\frac{E_{g}}{kT}\right)$$

where *k*, *T*, and *J*_0_ are the Boltzmann constant (1.38 × 10^−23^ J/K), thermodynamic temperature, and reverse saturation current, respectively.

Therefore, the *V_oc_* linearly increases with the bandgap of CIGS. On the contrary, *J_sc_* linearly decreases with the bandgap of CIGS (Figure 3b). The short-circuit current (*I_sc_*) is calculated according to the following equations [36]:

(6) $$I_{sc}=-I_{L}$$

(7) $$I_{L}=qAG\left(L_{e}+W+L_{h}\right)$$

Each photon reaching the solar cell surface with an energy greater than the bandgap of the absorption layer creates an electron-hole pair. Thus, the *J_sc_* (*J_sc_* = *I_sc_*/device area) decreases with the increase of the CIGS bandgap. The FF shows a quasi-linearly relationship with the bandgap of CIGS (Figure 3c). Under an ideal condition, the FF is only related to the *V_oc_* based on Equations (8) and (9) [37]:

(8) $${\mathrm{FF}}_{0}=\frac{v_{oc}-\ln\left(v_{oc}+0.72\right)}{v_{oc}+1}$$

(9) $$v_{oc}=V_{oc}\frac{q}{kT}$$

However, in a practical device, the FF is also affected by the series resistance (*R_s_*) and shunt resistance (*R_sh_*), leading to the quasi-linearly increase of the FF with the bandgap of CIGS. The PCE increases with the bandgap of CIGS when the *E*_g_ is lower than 1.40 eV (Figure 3d). When the bandgap of CIGS is larger than 1.40 eV, the PCE decreases with the increase of *E*_g_ (Figure 3d). The PCE of a solar cell is closely related to the *V_oc_*, *I_sc_*, and FF based on the following equation [38]:

(10) $$\mathrm{PCE}=\frac{V_{oc}\times J_{sc}\times FF}{P_{in}}\times 100\%$$

Therefore, based on the relationship between *V_oc_*, *J_sc_*, and FF and the bandgap of CIGS, the dependence of PCE on the bandgap of CIGS is easily achieved, as shown in Figure 3d. The PCE of the CIGS solar cell firstly increases and then decreases (Figure 3d) with the bandgap of CIGS in the region of 1.04–1.68 eV. Interestingly, the PCE is quite high in the whole bandgap region of CIGS, with a value of 20.57–24.55%. The maximum PCE is 24.55% when the CIGS bandgap is 1.40 eV. Thus, the CIGS bandgap is set to 1.40 eV for the following simulation.

**Figure 4:**
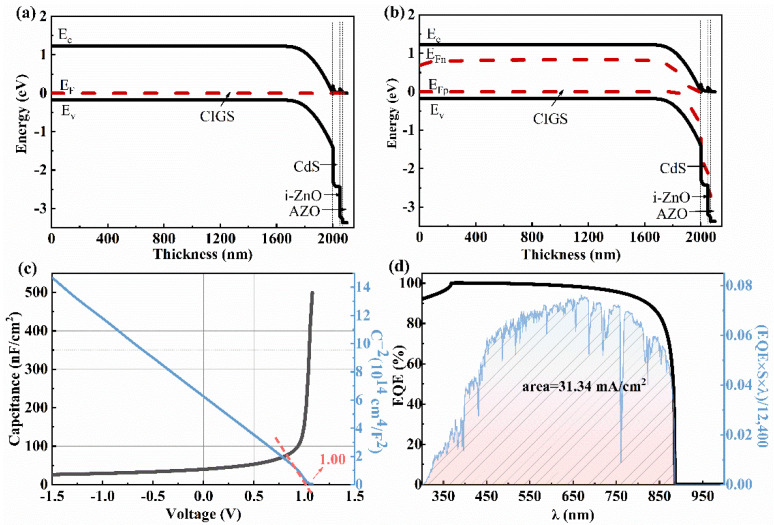
Energy band diagram of the CIGS (*E*_g_ = 1.4 eV) solar cell with a device structure of Mo/CIGS/CdS/i-ZnO/AZO under (**a**) dark and (**b**) AM1.5 G light illumination. (**c**) *C*-*V* and *C*^−2^-*V* curves, (**d**) EQE curve and the *J_sc_* value from the integral curve based on the expression $\frac{\mathrm{EQE}\times S\times \lambda }{12,400}$, λ is a wavelength of sunlight; S is the standard AM1.5 G spectrum.

Figure 4a,b shows the energy band diagrams of the CIGS solar cell with a CIGS bandgap of 1.40 eV under dark and AM1.5 G light illumination. Under light illumination, a spike-like conduction band offset (CBO) is observed at the interfaces of CIGS/CdS (~0.2 eV) and CdS/i-ZnO (~0.1 eV), implying a low interface carrier recombination [24]. According to the simulated *C*-*V* result (Figure 4c), the internal electric field is ~1.00 eV, consistent with the *V_oc_* of 931.70 mV obtained from the *I*-*V* curve (Table 2). The *V_oc_*_, *loss*_ (~0.40 eV) is probably due to the small width of the deletion region, determined from the energy band diagram under light illumination (Figure 4b). The low *J_sc_* mainly stems from the optical loss in the short wavelength (<418 nm) seen from the simulated EQE curve (Figure 4d). Thereby, the buffer layer, with a much wider bandgap than 2.45 eV of CdS, is required to reduce the optical loss and, thus, improve the PCE.

### 3.2. Impact of BTO Thickness on Performances of BTO-Coupled CIGS Devices

**Figure 5:**
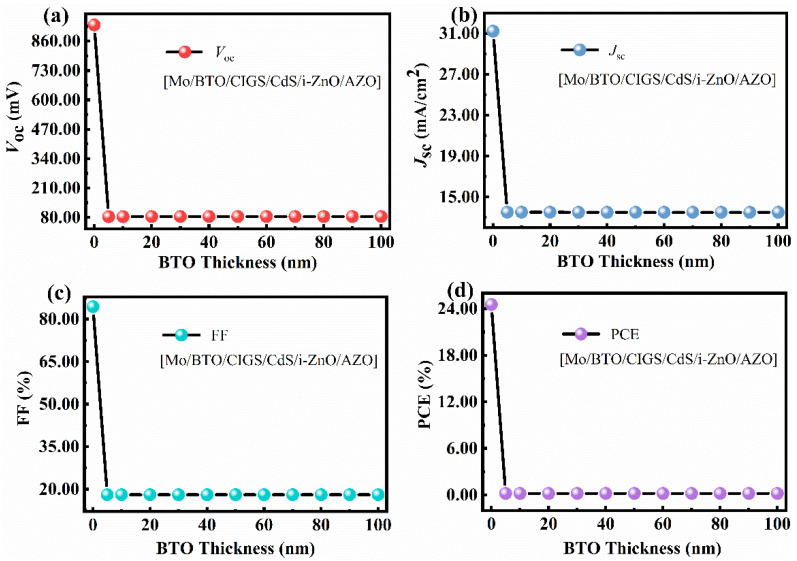
Dependence of (**a**) *V_oc_*, (**b**) *J_sc_*, (**c**) FF, and (**d**) PCE on the thickness of the BTO layer of the BTO-coupled CIGS solar cell with a device configuration of Mo/BTO/CIGS/CdS/i-ZnO/AZO.

**Figure 6:**
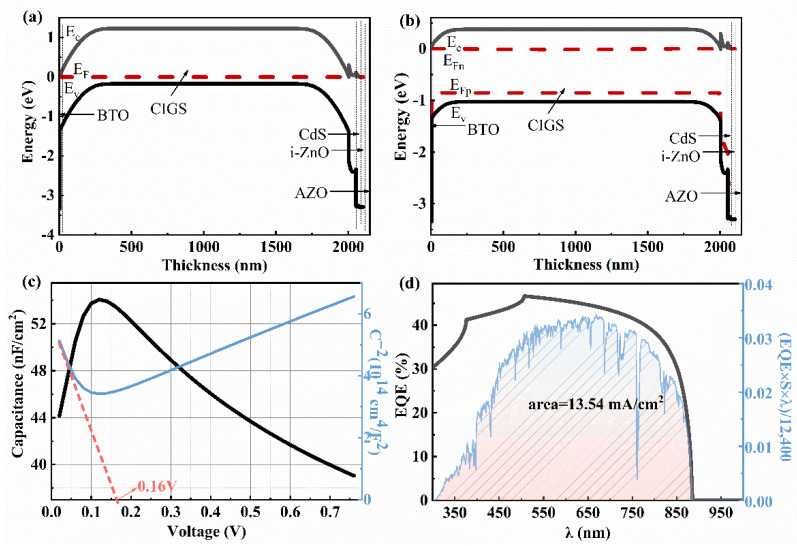
The energy band diagram, *C*-*V*, and EQE curves of the solar cell with a device structure of Mo/BTO/CIGS/CdS/i-ZnO/AZO and a CIGS bandgap of 1.40 eV: (**a**) under non-light conditions, (**b**) under the AM1.5 G, 1SUN, 1000 W/m^2^ condition, (**c**) *C*-*V* curve and *C*^−2^-*V* curve, (**d**) EQE curve and the *J_sc_* value from the integral curve based on the expression $\frac{\mathrm{EQE}\times S\times \lambda }{12,400}\;$.

In our study, the BTO was inserted into the different locations of the CIGS solar cell to clarify the impact of BTO on the performance of the CIGS solar cell, as shown in Figure 1. The internal electric field was expected to be enhanced because the BTO ferroelectric layer has a large depolarization field (*E_dp_*) after poling [39]:

(11) $$E_{dp}=\frac{d\sigma_{p}}{\varepsilon_{0\;}\varepsilon_{FE}L}$$

where *σ_p_* is the polarization charge density, *d* is the thickness of the BTO thin film, *L* is the thickness of the semiconductor layer, and *ε_FE_* is the relative dielectric constant of the BTO. The thickness of the BTO ferroelectric film should be very thin because the ultrathin ferroelectric layer has a large surface charge density. For this, the thickness of the BTO layer was set to be 5–100 nm. The simulated results show that the location of BTO has a significant effect on the *V_oc_*, *J_sc_*, FF, and PCE. The *V_oc_*, *J_sc_*, FF, and PCE decreased significantly when a BTO layer was inserted between the Mo and CIGS layers. The maximum PCE of the solar cell was quite low, only 0.20%, with a *V_oc_*, *J_sc_*, and FF of 0.08 V, 13.51 mA/cm^2^, and 18.05% (Figure 5 and Table 2). The device performance of the Mo/BTO/CIGS/CdS/i-ZnO/AZO was poor and nearly independent of the BTO thickness (Figure 5). The reason for the low PCE was due to the formed BTO/CIGS back field, as seen from the energy band diagram (Figure 6a,b). The electric field between the BTO/CIGS showed an opposite direction to that of the CIGS/CdS because of the *n*-type of the CdS and BTO. The Fermi level difference between *E*_fn_ and *E*_fp_ had nearly the same value (Figure 6b). Thus, the internal electric field strength between the BTO/CIGS and CIGS/CdS showed little difference, which may be due to the similar *n*-type nature and bandgap of the BTO (*E*_g_ = 3.4 eV) and AZO (*E*_g_ = 3.3 eV) [39]. The internal electric field, calculated from the *C*-*V* curve (Figure 6c), was only 0.16 V [40], leading to a very low *V_oc_*. The integrated *J_sc_* from the EQE curve (Figure 6d) was 13.54 mA/cm^2^, in good agreement with the *J_sc_* of 13.51 mA/cm^2^ obtained from the *I*-*V* result (Figure 5b and Table 2). The same capability in the separation and transportation of carriers in the opposite directions of the BTO/CIGS and CIGS/CdS/i-ZnO/AZO led to the weak separation and transportation of light-induced carriers, resulting in the low *J_sc_* (Figure 5b). The low *V_oc_* resulted in a low FF. The low *V_oc_*, *J_sc_*, and FF resulted in the low PCE of the solar cell. Thereby, it is seen that the BTO located between the CIGS and Mo layers significantly reduced the device’s performance.

**Figure 7:**
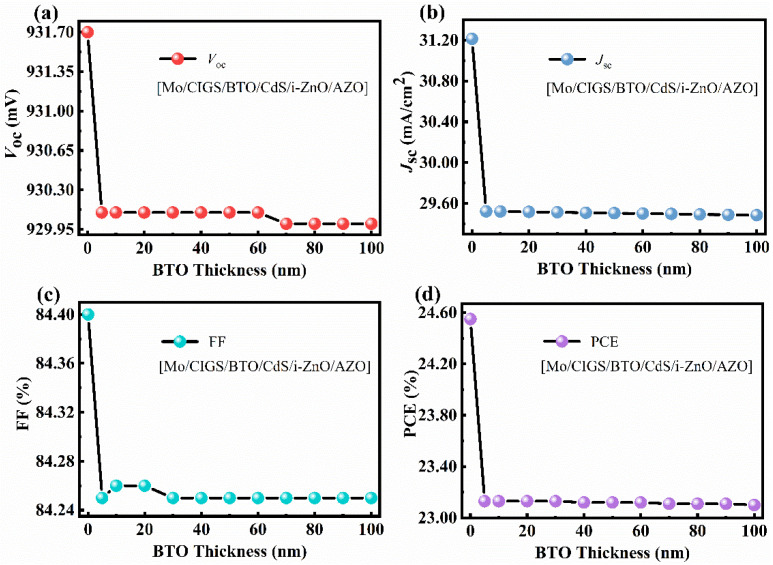
Dependence of (**a**) *V_oc_*, (**b**) *J_sc_*, (**c**) FF, and (**d**) PCE on the thickness of the BTO in the ferroelectric-coupled CIGS solar cell with a device structure of Mo/CIGS/BTO/CdS/i-ZnO/AZO.

**Figure 8:**
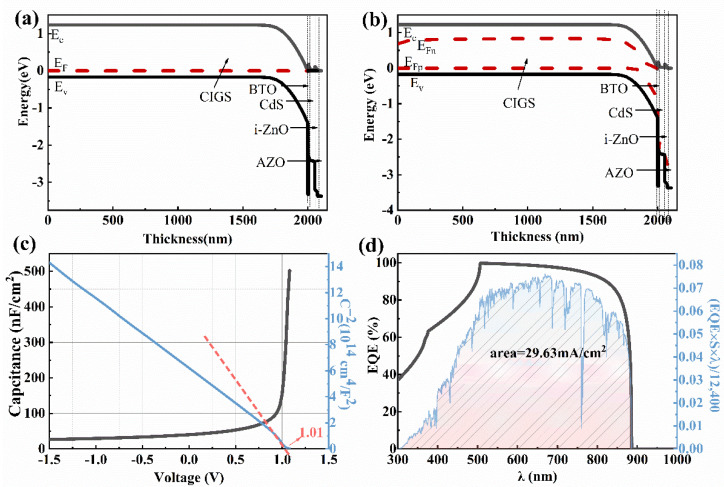
The energy band diagram, *C*-*V*, and EQE curves of the ferroelectric-coupled CIGS solar cell with a device structure of Mo/CIGS/ BTO/CdS/i-ZnO/AZO and a CIGS bandgap of 1.40 eV: (**a**) under non-light conditions, (**b**) under the AM1.5 G, 1SUN, 1000 W/m^2^ condition, (**c**) *C*-*V* curve and *C*^−2^-*V* curve, (**d**) EQE curve and the *J_sc_* value from the integral curve based on the expression $\frac{\mathrm{EQE}\times S\times \lambda }{12,400}$.

Based on the simulated results, the performance of the CIGS solar cell is expected to be improved when the BTO is moved onto the top of the *p*-type CIGS because of the *n*-type and ferroelectric nature of the BTO. As shown in Figure 7, the performance of the device still shows little dependence on the thickness of the BTO layer. The *V_oc_*, *J_sc_*, FF, and PCE of the device with a device structure of Mo/CIGS/BTO/CdS/i-ZnO/AZO are greatly improved when the BTO is moved onto the top of CIGS (Figure 7). Importantly, the *V_oc_* increases from 83.60 mV to 930.10 mV. The enhanced *V_oc_* is attributed to the enhanced strength of the internal electric field. The strength of the internal electric field, calculated from the *C*-*V* curve, is 1.01 V (Figure 8c). The enhanced internal electric field is largely due to the change of the energy band diagram of Mo/CIGS/BTO/CdS/i-ZnO/AZO compared with that of Mo/BTO/CIGS/CdS/i-ZnO/AZO, as shown in Figure 8a,b. Clearly, the energy band diagram at the back contact is greatly changed, showing that the location of the BTO greatly affects the internal electric field. The energy band diagram shows a similar character to that of the CIGS solar cell with a device structure of Mo/CIGS/CdS/i-ZnO/AZO (Figure 4), indicating the enhanced internal electric field. The enhanced internal electric field improves the *V_oc_* and the separation and transportation of the light-induced carriers, which contributes to the enhanced *J_sc_*. The integrated *J_sc_* from the EQE curve (Figure 8d) is 29.63 mA/cm^2^, which is in good agreement with the *J_sc_* of 29.52 mA/cm^2^ obtained from the simulated *J*-*V* curve (Figure 7b and Table 2). The enhanced *V_oc_* leads to the enhanced FF. The improved *V_oc_*, *J_sc_*, and FF thus enhance the PCE of the solar cell. The maximum PCE is 23.13% with a *V_oc_*, *J_sc_*, and FF of 930.10 mV, 29.52 mA/cm^2^, and 84.25% (Figure 7 and Table 2) when the thickness of BTO is 5 nm.

**Figure 9:**
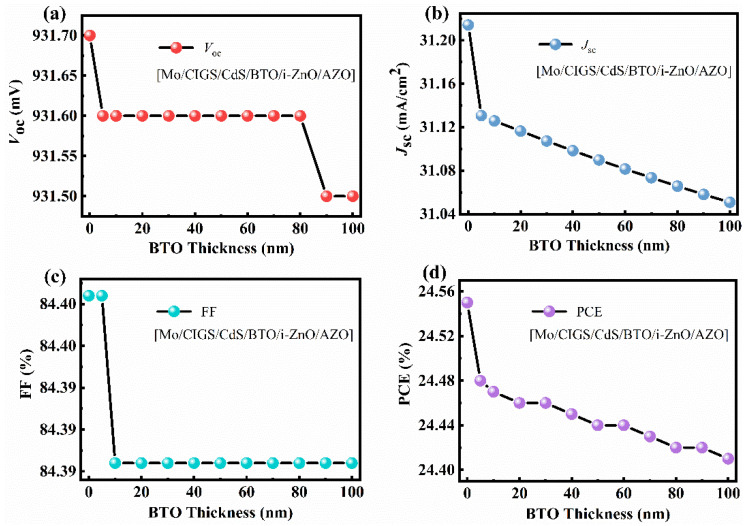
Effect of the BTO thickness on the (**a**) *V_oc_*, (**b**) *J_sc_*, (**c**) FF, and (**d**) PCE of the CIGS solar cell with a device structure of Mo/CIGS/CdS/BTO/i-ZnO/AZO.

**Figure 10:**
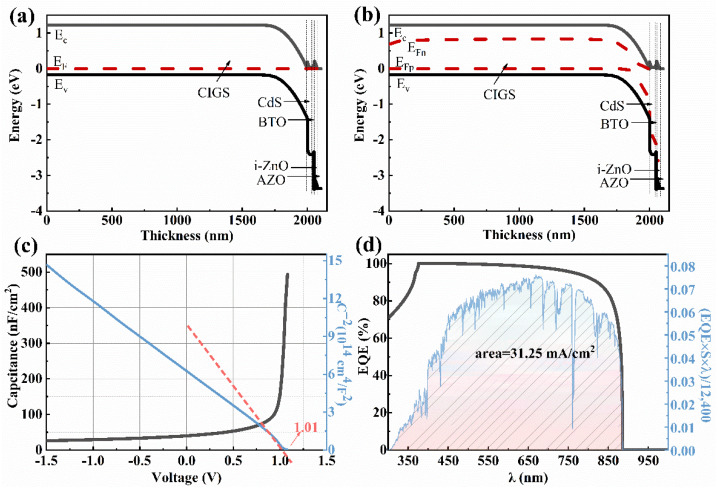
The energy band diagram, *C*-*V*, and EQE curve of the ferroelectric-coupled CIGS solar cell with a device structure of Mo/CIGS/CdS/BTO/i-ZnO/AZO: (**a**) under dark, (**b**) under the AM1.5 G light illumination, (**c**) *C*-*V* curve and *C*^−2^-*V* curves, and (**d**) EQE curve and the *J_sc_* value from the integral curve based on the expression $\frac{\mathrm{EQE}\times S\times \lambda }{12,400}$.

**Figure 11:**
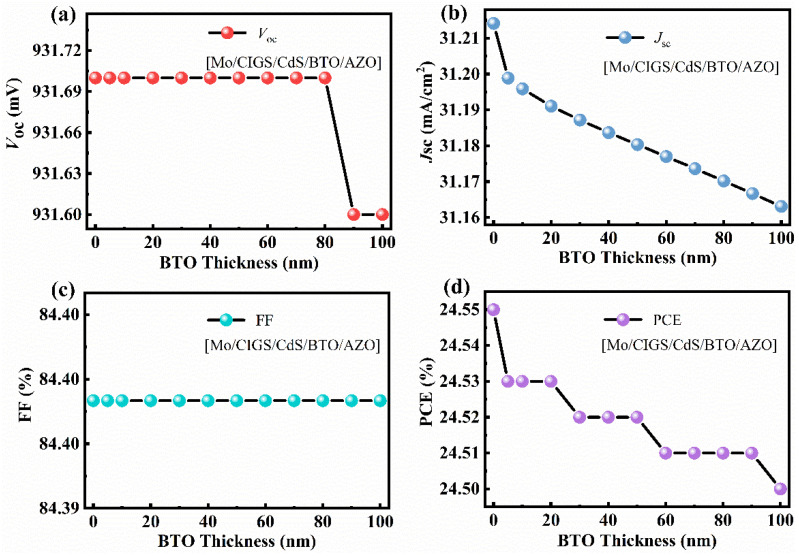
Effect of the BTO layer thickness on the electrical parameters of the ferroelectric-coupled CIGS solar cell with a device structure of Mo/CIGS/CdS/BTO/AZO: (**a**) *V_oc_*, (**b**) *J_sc_*, (**c**) FF, and (**d**) PCE.

**Figure 12:**
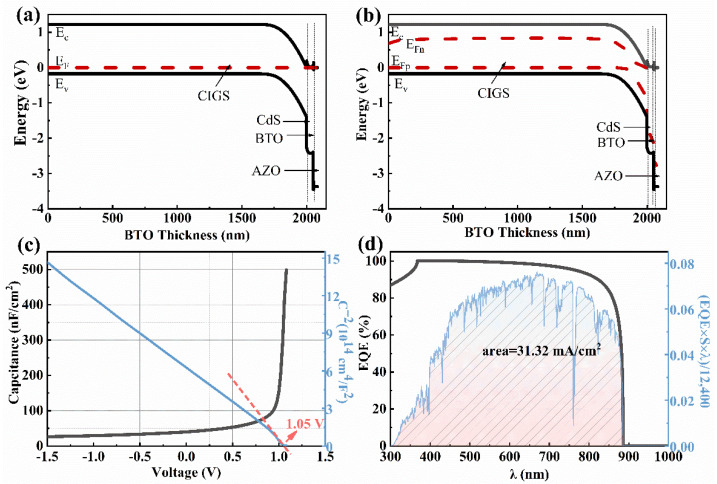
The energy band diagram, *C*-*V*, and EQE curves of the ferroelectric-coupled CIGS solar cell with a device structure of Mo/CIGS/CdS/BTO/AZO: (**a**) under non-light conditions, (**b**) under the AM1.5 G light illumination, (**c**) *C*-*V* and *C*^−2^-*V* curves, (**d**) EQE curve and the *J_sc_* value from the integral curve based on the expression $\frac{\mathrm{EQE}\times S\times \lambda }{12,400}$.

As shown in Figure 9a, when the BTO thickness is 5–80 nm, the *V_oc_* improves from 930 mV to 932 mV when the BTO layer is on the top of CdS. Although the *V_oc_* decreases with the increase of the BTO thickness when the thickness of the BTO is larger than 80 nm, the *V_oc_* is still higher than that of the device with an architecture of Mo/CIGS/BTO/CdS/i-ZnO/AZO. The decrease of the *V_oc_* with the thickness of the BTO is due to the large surface charge density at the ultrathin ferroelectric layer [39]. As seen in Figure 10c, the internal electric field is 1.05 V, which is higher than that of 1.01 V for the device with a structure of Mo/CIGS/BTO/CdS/i-ZnO/AZO. The increased internal electric field results in the increased *V_oc_*. The energy band diagram, especially the valence band, becomes much more suitable for the separation and transport of the light-induced carriers (Figure 10a,b) and thus leads to the enhanced *J_sc_*. The *J_sc_* linearly decreases with the BTO thickness (Figure 9b), which attributes to the optical absorption of the BTO. The maximum *J_sc_* is 31.25 mA/cm^2^ (Figure 10d). The FF shows a similar dependence of the BTO thickness with that of *V_oc_* (Figure 9c), which can be well explained based on Equations (8) and (9). Thus, the PCE displays a similar relationship with that of *J_sc_* between the BTO thickness (Figure 9d), which is well explained according to Equation (10). The maximum PCE of the solar cells is 24.48%, as listed in Table 2. Thereby, it is further confirmed that the BTO location greatly affects the performance of the ferroelectric-coupled CIGS solar cells. As shown in Table 2, the PCE, *V_oc_*, and *J_sc_* of the solar cell show nearly no change when the CdS is removed. Interestingly, the electric parameters and the energy band diagram of the devices also show nearly no change when the i-ZnO is removed from the device (Figure 11 and Figure 12). The highest PCE of the device with a 5 nm BTO layer is increased to 24.53% (Table 2 and Figure 11d), which is mainly due to the much flatter valence band offset (Figure 12b) when compared to that of the ferroelectric-coupled CIGS solar cell with a device structure of Mo/CIGS/CdS/BTO/i-ZnO/AZO (Figure 10b). The maximum *J_sc_* is 31.20 mA/cm^2^ when the thickness of the BTO is 5 nm (Figure 11b). The *J_sc_* is slightly higher than the *J_sc_* of 31.12 mA/cm^2^ for the device with a configuration of Mo/CIGS/CdS/BTO/i-ZnO/AZO. The slightly increased *J_sc_* is owed to the removal of the parasitic optical absorption layer of i-ZnO.

**Figure 13:**
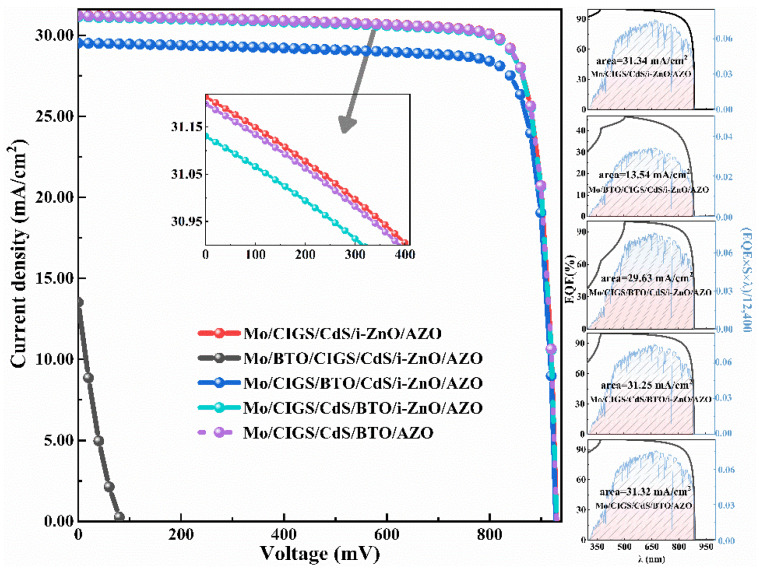
The *J*-*V*, corresponding EQE, and the integrated EQE curves for the different devices with the highest simulated PCE.

Thereby, it is concluded that the ferroelectric-coupled CIGS solar cell is a promising solar cell. The *J*-*V*, corresponding EQE, and the integrated EQE curves for the devices with the highest simulated PCE are shown in Figure 13. The corresponding device parameters are listed in Table 2 in detail. The performance of this device is influenced little by the thickness of the CdS when the CdS thickness is 5–50 nm (Table 2), showing the potential low fabrication cost of the solar cells thanks to less material usage.

Thus, the optimized device configuration is Mo/CIGS/CdS/BTO/AZO with a 5 nm BTO. The thinner BTO is beneficial to the carries tunnel and provides a stronger ferroelectric field. The PCE for this device is 24.53% with a *V_oc_* of 931.70 mV, which is comparable to the PCE of 24.55% for the CIGS solar cell with a typical device configuration of Mo/CIGS/CdS/i-ZnO/AZO (Figure 13 and Table 2). The PCE for the device with a device configuration of Mo/CIGS/CdS/BTO/i-ZnO/AZO is 24.48% with a *V_oc_* of 931.60 mV.

**Figure 14:**
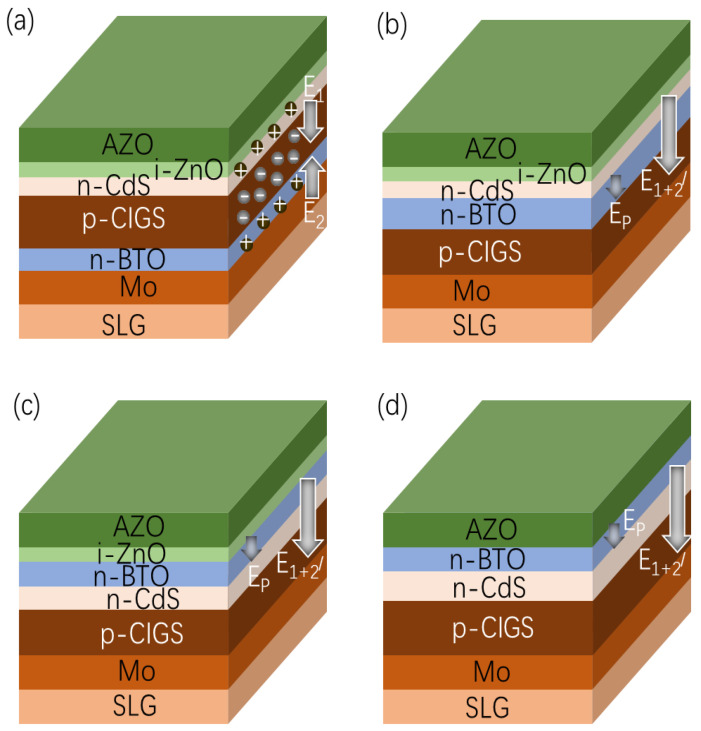
The different device structures of CIGS cells (not drawn to scale) and the schematic diagram of the electric field induced by the polarized BTO thin film and the field-assisted carrier separation. (**a**) SLG/Mo/BTO/CIGS/CdS/i-ZnO/AZO/Au, (**b**) SLG/Mo/CIGS/BTO/CdS/i-ZnO/AZO/Au, (**c**) SLG/Mo/CIGS/CdS/BTO/i-ZnOAZO/Au, and (**d**) SLG/Mo/CIGS/CdS/BTO/AZO/Au.

The working mechanism for the CIGS solar cells with different device structures is shown in Figure 14. When the BTO is located between Mo and CIGS, a *p*-*n* junction field is formed between the BTO and CIGS. The *p*-*n* junction electric field between the BTO/CIGS has an opposite direction to that of the CIGS/CdS (Figure 14a), which leads to the low PCE of the solar cells. However, the *p*-*n* junction electric field between the BTO and CIGS shows the same direction as the CIGS/CdS *p*-*n* junction electric field (Figure 14b) when the BTO is between the CIGS and CdS. When the BTO is poled, the depolarization field also shows the same direction as that of the built-in *p*-*n* electric field. Therefore, the strength of the total field is enhanced greatly. After the BTO is moved to the top of the CdS, the CIGS/CdS *p*-*n* junction electric strength is not affected by the insertion of the BTO. When the BTO is poled, the depolarization field shows the same direction as the built-in *p*-*n* electric field (Figure 14c). The total electrical field is thus enhanced, leading to an enhanced performance of the device. For the device with a structure of SLG/Mo/CIGS/CdS/BTO/AZO/Au (Figure 14d), the CIGS/CdS *p*-*n* junction electric strength is not affected by the removal of i-ZnO; thus the coupled *p*-*n* junction electric field and depolarization electric field can be retained. Interestingly, the parasitic optical absorption is reduced by the removal of the i-ZnO, resulting in the slightly enhanced PCE of the device.

## 4. Conclusions

In this study, BTO-coupled CIGS solar cells with various structures have been systematically investigated via the SCAPS-1D software. The performance of the CIGS solar cell was closely related to the location of the BTO layer. The CIGS solar cell had the maximum PCE when the BTO layer was on the top of the *p*-*n* junction. The effect of the BTO thickness on the performance of the CIGS solar cell was also studied. The optimized device configuration was Mo/CIGS/CdS/BTO/AZO, with a 5 nm-thick BTO. The PCE of this device was 24.53% with an FF of 84.40%, a *J_sc_* of 31.19 mA/cm^2^, and a *V_oc_* of 931.70 mV.

## Figures and Tables

**Table 1:** Input simulation parameters for different layers in CIGS and BTO-coupled CIGS solar cells [31,32,33].

Parameters	*p*-CIGS	*n*-CdS	i-ZnO	AZO	BTO
	Absorber	Buffer	Window		Ferroelectric
Thickness (nm)	2000	5–50	20	30	5–100
Bandgap *E*_g_ (eV)	1.04–1.68	2.45	3.30	3.37	3.40
Electron affinity *χ* (eV)	4.6	4.4	4.3	4.3	4.5
Relative dielectric permittivity *ε_r_*	13.6	10.0	9.0	9.0	290.0
Effective conduction band density *N_c_* (cm^−3^)	6.8 × 10^17^	1.3 × 10^18^	3 × 10^18^	1 × 10^20^	4 × 10^18^
Effective valence band density *N_v_* (cm^−3^)	1.5 × 10^19^	9.1 × 10^18^	1.7 × 10^19^	3 × 10^18^	9 × 10^18^
Electron thermal velocity *ν_n_* (cm/s)	1 × 10^7^	3.1 × 10^7^	1 × 10^7^	1 × 10^7^	1 × 10^7^
Hole thermal velocity *ν_p_* (cm/s)	1 × 10^7^	1 × 10^7^	1 × 10^7^	1 × 10^7^	1 × 10^7^
Electron mobility *μ_n_* (cm^2^/(Vs))	100	72	100	100	50
Hole mobility *μ_p_* (cm^2^/(Vs))	12.5	20	31	31	20
Donor concentration *N_D_* (cm^−3^)	0	5 × 10^17^	1 × 10^17^	1 × 10^20^	5 × 10^17^
Acceptor concentration *N_A_* (cm^−3^)	2 × 10^16^	0	0	0	0
Defect density (cm^−2^)	5 × 10^13^	3 × 10^13^	1 × 10^16^	3 × 10^16^	0

**Table 2:** The simulated device parameters for PV devices with different device architectures.

Device Architectures	*V_oc_* (mV)	*J_sc_* (mA/cm^2^)	FF (%)	PCE (%)
Mo/CIGS/CdS/i-ZnO/AZO	931.70	31.21	84.40	24.55
Mo/BTO/CIGS/CdS/i-ZnO/AZO	83.60	13.51	18.05	0.20
Mo/CIGS/BTO/CdS/i-ZnO/AZO	930.10	29.52	84.25	23.13
Mo/CIGS/CdS/BTO/i-ZnO/AZO	931.60	31.13	84.40	24.48
Mo/CIGS/BTO/i-ZnO/AZO	931.60	31.13	84.34	24.46
Mo/CIGS/CdS/BTO/AZO	931.70	31.19	84.40	24.53
Mo/CIGS/CdS(5 nm)/BTO(5 nm)/i-ZnO/AZO	931.60	31.12	84.33	24.45

(These data are obtained under AM1.5 G, 1SUN, 1000 W/m^2^ light illumination conditions.)

## Data Availability

Not applicable.
